# Cancer survivors’ experiences of using survivorship care plans: a systematic review of qualitative studies

**DOI:** 10.1007/s11764-014-0407-x

**Published:** 2014-10-25

**Authors:** Sharon Keesing, Beverley McNamara, Lorna Rosenwax

**Affiliations:** School of Occupational Therapy and Social Work, Curtin University, Perth, Western Australia

**Keywords:** Survivorship care plans, Experiences, Long-term, Qualitative research

## Abstract

**Purpose:**

Cancer survivorship care plans (SCPs) are currently used in care settings to assist survivors during the transition from treatment to survivorship. In this paper, the experiences of cancer survivors are examined to provide their perspective of how survivorship care plans are used in practice.

**Methods:**

A systematic review and critical review of the qualitative literature regarding the experiences of cancer survivors using survivorship care plans was completed. Databases reviewed included CINAHL, AMED, Embase, MEDLINE, Informit, ProQuest, PsycINFO, ScienceDirect, Wiley Online Library, Scopus and Web of Science from 2000 to 2014.

**Results:**

Eleven qualitative studies were appraised for methodological quality and content. They revealed four key themes: stakeholders agreed that SCPs should be used as a key strategy for cancer survivors; there was a lack of consensus on the format, content and who should develop the SCP; cancer survivors do not consistently receive SCPs; and there was a lack of evidence to support the use of SCPs in practice.

**Conclusions:**

There is great potential for SCPs to assist cancer survivors and this is supported by the range of qualitative literature examined in this study. Further research is required to examine the many practical issues relating to the delivery of SCPs and how they may be used across a variety of care contexts as well as providing further evidence to support their use.

**Implications for Cancer Survivors:**

With further research, refinement and contributions made by survivors, health researchers and health care professionals, the survivorship care plan is proposed to be a useful and practical tool aimed at supporting the survivorship continuum of care.

## Introduction

The proportion of people surviving cancer is increasing in many parts of the world due, in part, to early diagnosis, increased rates of detection and significant improvements in treatment [1–3]. However, many cancer survivors experience a range of ongoing difficulties related to the disease itself and the treatments used. These difficulties continue to impact upon survivors during, immediately after and, for some individuals, many years after completion of their treatment [4–6]. Well-recognised physical problems include chronic pain, weight gain, osteoporosis, premature menopause and memory and sleep disturbances. Additional consequences of cancer and cancer treatments can contribute to further difficulties including a range of chronic health conditions including heart disease and diabetes [7]. Psychological difficulties including depression and anxiety may also be experienced, and these may be associated with ongoing symptoms of fatigue, sexual dysfunction, fear of recurrence and changes to relationships [8, 9]. A recent Australian study identified that long-term survivors of cancer reported increased levels of vulnerability, loneliness and anxiety about their health and the possibility of the cancer returning [10]. It is evident that many cancer survivors experience significant ongoing problems with resuming their usual roles and relationships as well as returning to their previous routines and habits.

There are many less-recognised and sparsely publicised issues for survivors following cessation of treatment including social difficulties, maladjustment to work responsibilities, intimacy problems, organisational difficulties and cognitive processing issues [11–13]. The literature also identifies a range of existential problems affecting cancer survivors including challenges pertaining to self-identity and personal expectations [14]. Globally, survivors are increasingly seeking a wider range of supports and services during the post-treatment period to assist them with the breadth of physical and psychological difficulties experienced in the longer term [13, 15, 16]. In most developed countries, a range of strategies are currently available to assist cancer survivors with these ongoing difficulties, including access to health services, support groups, online forums and educational tools. However, it appears that there is a lack of recognition of this period with referral and coordination for follow-up care needed during cancer survivorship [6].

The Survivorship care plan (SCP) is postulated as a potential resource to improve survivorship care. The SCP is recognised as an important tool that may be used during the survivorship period and one which is attracting further research in the international context because of its potential to assist survivors to direct and navigate their own ongoing care [8, 10, 13, 17–19].

## Use of survivorship care plans across the world

Researchers in the United States of America (USA), Canada and the United Kingdom (UK) have made significant contributions to the study of the survivorship phenomenon [4]. In 2005, the Institute of Medicine (IOM) (USA) published its report ‘From cancer patient to cancer survivor: lost in transition’ [8]. This report outlined key recommendations to assist cancer survivors in the longer term including the use of SCPs. The SCP usually includes a summary of diagnosis and treatment, methods of surveillance for the potential development of malignancies, maintenance of healthy lifestyle, legal and financial rights and identification of support services [20]. These recommendations have been widely accepted in many countries as critical to the care of survivors yet have not been fully evaluated. In a randomised control trial conducted by Grundfeld [21], it was found that there was little evidence to support the use of the SCP in practice. However, this study was limited to survivors of breast cancer and its generalisability to other types of cancer, as well as the context of care, has not been investigated [22].

Canada has had a national cancer strategy since 2007 which identifies key priorities for survivorship: the development and implementation of national standards and models of care; promotion of survivorship research, knowledge and communication plans and advocacy groups; and an emerging interest in the use of SCPs [23]. In the UK, the National Cancer Survivorship Initiative (NCSI) articulates the care of survivors using a recovery-focussed, personalised approach with explicit outcome measures to determine the effectiveness of the services provided. It recommends that all survivors are offered a treatment summary and care plan as well as appropriate education and information [4].

Australia does not have a national cancer plan or consistent model of care for cancer survivors. Models of care across the states of Australia vary and include the disease-specific model, general survivorship model, consultative clinic, multidisciplinary clinic, integrated care model and transition to primary care model [24]. This has resulted in each state providing a different framework of care, a variety of practice guidelines and a range of state-based services. According to one Australian author, Jefford et al. [4], the ‘traditional’ medical follow-up currently offered may not meet survivors’ needs due to its focus on cancer recurrence and not on other important, but less obvious, concerns. The SCP which offers a summary of treatment, surveillance and recommendations for follow-up care is a key resource used in many other countries, however, to date, not used consistently in Australia and other countries.

There is a range of published literature that explores the use of SCPs in many different countries and contexts of care with sources of input from oncologists, primary care physicians/general practitioners and oncology nurses. More recently, systematic reviews of the quantitative literature have been conducted that consider a range of issues including survivorship models of care [25] and the effectiveness of the SCP [3]. While these studies contribute to the range and breadth of the literature, there has been very little consideration of survivors’ own perspectives regarding their experiences and views about the use of the SCP [20, 26, 27]. As SCPs are used by a range of health professionals, it is essential that the preferences of survivors, as key stakeholders in the continuum of care, are explored and articulated to ensure services and supports are directed appropriately.

The aim of this systematic review is to document and review the available published qualitative literature that describes cancer survivors’ experiences of using survivorship care plans.

## Methods

An examination of existing published systematic reviews and protocols was undertaken to determine whether the research question was appropriate due to previously published literature on the subject. The following sources were initially reviewed: Cochrane Collaboration, Joanna Briggs Institute, Database of Abstracts of Reviews of Effects (DARE), PROSPERO and Trip. It was established that no existing systematic reviews of the research question had been registered or published to date.

## Search strategy and data sources

A protocol for searching was established prior to commencement of the search. Priori inclusion criteria were determined as follows: adults only (18 years and above), date range from 2000 to 2014 and published in English. Publications were excluded if they reported on the experience of cancer treatment or the palliative phase of care and were conference presentations or abstracts only. Searches of electronic databases completed were CINAHL, AMED, Embase, MEDLINE, Informit, ProQuest, PsycINFO, ScienceDirect, Wiley Online Library, Scopus and Web of Science. The *Journal of Cancer Survivorship* was also reviewed for publications that met the inclusion criteria. Permutations of the following search terms were truncated and exploded: cancer, neoplasm or malignancy survivor, experiences, opinions, ideas, views or preferences, survivorship care plan, post-treatment care, forward care, survivorship program, individualised care plan and comprehensive care plan.

**Fig. 1:**
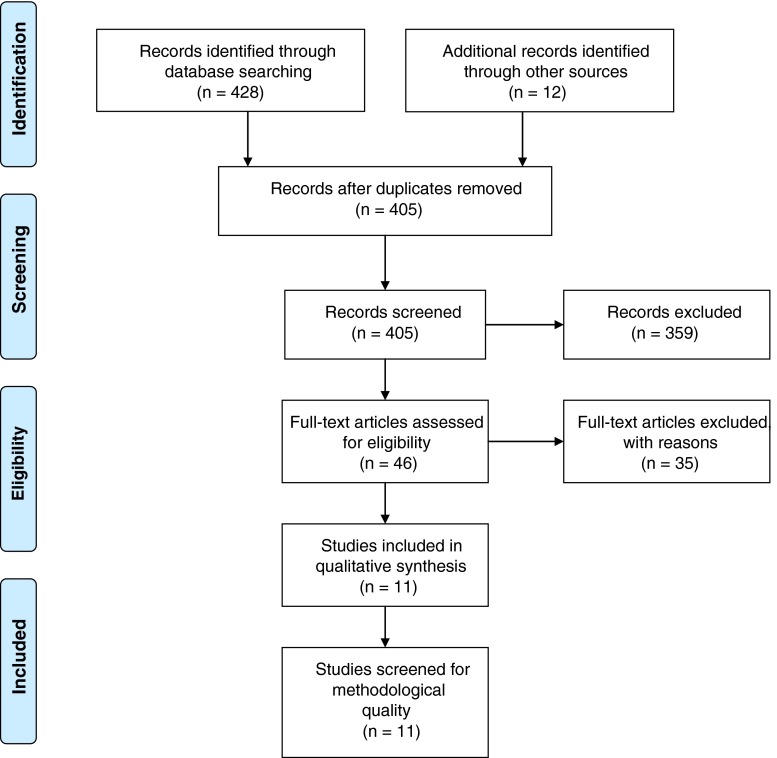
Data screening flow chart

The searches identified a total of 428 records using the search terms above. Hand searching of reference lists identified an additional 12 articles for review. Thirty five records were excluded as duplicates. The title and abstracts of the remaining 405 records were reviewed by the first author and 46 of these met the prior inclusion criteria for full review. The full-text articles were then reviewed independently by the first and second authors using the eligibility criteria of any of the following: qualitative studies including systematic reviews, interviews, focus groups, case studies, descriptive studies, observational and narrative studies; action research as well as the qualitative components of mixed methods studies. Additional eligibility criteria included survivors’ experiences of using a SCP and analysis and discussion about the findings of the study (refer to Fig. 1 for details of data screening). In total, 11 papers were determined as eligible for critical appraisal of methodological quality.

## Data analysis

Critical appraisal of methodological quality was completed using the ‘Standard Quality Assessment Criteria for Evaluating Primary Research Papers from a Variety of Fields’ by Kmet, Lee and Cook [28]. This tool uses a numeric score (0–2) to rate the quality of ten categories considered essential to the research credibility. The categories assessed are as follows: research question or objective stated clearly, explanation of the study design, context clearly defined, connection to a theoretical framework, justification of the sampling strategy, description of data collection, clearly defined analysis of results, use of verification procedures discussed, conclusions drawn and reflexivity of authors considered. The first two listed authors independently assessed each paper using these criteria to determine a total score out of a possible 20. Where there was a difference in scores of greater than 1/20, discussion and consensus agreement was reached. The calculated scores were defined according to quality as strong (score of >80 %), good (70–80 %), adequate (50–70 %) or limited (<50 %). Studies were included if they received a quality score of 70 % and above. All of the 11 studies reviewed met the minimum requirement of 70 % on the assessment criteria. A list of each study’s scores using the appraisal tool can be found in Table 1. The total number of participants included across all reviewed studies was 336 with one study not stating the number of participants.

**Table 1:** Demographic data of all studies

Author and year	Population and gender	No. of participants	Location	Age range (years)	Time since diagnosis	Time since completion of treatment	Cancer type	Type of study	SCP used	Experiences of survivorship?	Methodological quality using KMET/20
Ashing-Giwa et al. 2013 [29]	African-American women	28	USA	31–80	0–10 years	Not stated	Breast	Community-based participatory framework	Yes	Yes	16
Baravelli et al. 2009 [30]	Australian men	12	Australia	47–77	Not stated	Not stated	Bowel	Interviews and questionnaire	Yes	Yes	14
Brennan et al. 2011 [31]	Australian women	20	Australia	40–59	Range <2 to >10 years	Not stated	Breast	Interviews	Yes	Yes	17
Burg et al. 2009 [32]	African-American, Hispanic, Caucasian women	32	USA	18–65	Not stated	Not stated	Breast	Focus groups	Yes	Yes	18
Faul et al. 2012 [33]	American/not stated	7 survivors 7 oncology providers	USA	54–84	Not stated	Average 18 months	Colorectal	Interviews	Yes	Yes	15
Hewitt et al. 2007 [34]	American/men and women	36	USA	25–70	Not stated	Less than 5 years	All except skin cancer	Focus groups	Yes	Yes	14
Kantsiper et al. 2009 [35]	American women	21	USA	Not stated	Range <5 to >10 years	Not stated	Breast	Focus groups	Yes	Yes	18
Marbach et al. 2011 [36]	American men and women	40	USA	Not stated	Not stated	Not stated	Range	Focus groups	Yes	Yes	14
Parry et al. 2011 [37]	American men and women	51	USA	20–82	Not stated	3–48 months	Mostly lymphoma and leukaemia	Interviews	Yes	Yes	18
Singh- Carlson et al. 2013 [38]	Southeast Asian women	24	Canada	<44 to >65	Not stated	4–27 months	Breast	Focus groups	Yes	Yes	20
Smith et al. 2011 [39]	Canadian women	26	Canada	45–80	Not stated	3–12 months	Breast	Focus groups	Yes	Yes	16

## Summary of results

The 11 studies included the use of interviews (four studies), focus groups (six studies) and action research (one study). Table 1 provides a comprehensive summary of each study including sample location, population, number of participants, gender, age range, cancer type, time since diagnosis, time since completion of treatment and marital/partnership status. A content analysis was undertaken to review and understand the breadth and depth of the themes discussed for each of the studies by each of the researchers [40]. These themes were discussed and refined and are presented as follows.

## Data analysis and synthesis of results

Four significant themes were identified following content analysis of the 11 articles: (1) stakeholders agree that SCPs should be used as a key strategy for cancer survivors; (2) lack of consensus on what the SCP should contain and who should develop it; (3) cancer survivors do not consistently receive a SCP and (4) there was a lack of evidence to support the use of SCP in practice.

### Stakeholders agreed that SCPs should be used as a key strategy for cancer survivors

The SCPs were identified as a key strategy during the post-treatment period for cancer survivors [29–39] by the authors of all studies. Significant points raised by researchers include the use of SCPs to reduce duplication of materials improved coordination of care and increased communication between health professionals and cancer survivors. Specific areas for improvement were that the SCP needed to be accessible and nontechnical and directed to address the cultural issues specific to particular groups of people.

A significant finding from the majority of authors [28, 30, 31, 34–37] was the recommendation that SCPs should be targeted to provide coordinated, individualised and patient-centred care. This was also the expectation of cancer survivors. There appeared to be many barriers preventing this occurring in practice due to the limitations of the time needed to complete them, the need for resources and a lack of training on how to complete them [30–32, 35, 36, 40].

The use of SCPs assisted cancer survivors to translate information from specialist providers to their primary care providers and gave direction for the future [31, 32, 34, 35, 39]. It was also noted that SCPs reduced duplication of information and helped to synthesise treatment information to provide patients with peace of mind, a written synopsis of treatment and a targeted surveillance strategy during follow-up [34, 38, 39]. Furthermore, a range of unique survivorship issues relating to cultural background were identified, and many authors stated the importance of considering these broader issues as part of the development of the SCP [29, 32, 35]. Ashing-Giwa et al. [29] and Burg et al. [32] noted the explicit concerns of African-American women survivors of breast cancer and discussed the importance of including resources to address questions regarding treatment-related skin pigment changes and the availability of genetic testing for family members. Singh Carlson et al. [38] noted concerns raised in their study regarding South Asian women living in Canada, including the significance of family relationships and importance of faith during and after the treatment period.

The use of patient-centred SCPs was thought to assist in the transition from treatment to survivorship but also needed to be used in conjunction with suitable models of care [31, 34, 37]. Many different models are used by cancer survivors including shared care, consultative care, the chronic illness model and transitional care which resulted in the use of a range of tools and strategies as well as the involvement of many health professionals. At times, the complexities of these models resulted in a breakdown in communication and coordination of care. Several authors commented that SCPs could be used as a resource to facilitate well-timed support and case coordination [32, 34, 36, 39].

### Lack of consensus on what the SCP should contain, what format it should follow and who should develop it

The studies provided a range of findings regarding three important issues: what to include in the SCP, the format of the SCP and who should be responsible for developing it. Four studies indicated the essential components of the SCP should be diagnostic and treatment summaries, side effects of treatment and signs and symptoms of recurrence [30, 32, 38, 39]. Two studies [34, 38] concluded that SCPs must not only consist of a generic template of key considerations but also include sections for personalised items relating specifically to the individual. These additional items included educational resources regarding lifestyle changes, nutrition, exercise and details of support organisations.

One author, Faul et al. [33], stipulated the need for SCPs to include a ‘designated key provider’ to assist with the transition between care environments and services. This was supported by Brennan et al. [31], who reported that the SCP could be used to improve care and coordination of key stakeholders during the survivorship period. Marbach et al. [36] indicated that an overview of late and long-term effects also needed to be included as well as referrals for health professional services.

A significant finding from ten of the 11 reviewed studies was that currently, SCPs do not identify or address the significant psychosocial needs reported by cancer survivors [29–32, 34–39]. Both Burg et al. and Kantisper et al. reported that the breast cancer survivors in their studies had specific concerns regarding the need for assistance regarding an altered body image, breast reconstruction issues and weight gain [32, 35]. Depression, fear of recurrence and difficulties with relationships, intimacy and sexual function were described by Singh-Carlson et al. [38]. A ‘sense of abandonment’ as survivors transitioned from the treatment phase to survivorship was also discussed by Parry et al. [37] and Burg et al. [32] who explained this period as being pivotal for the adjustment between these two periods. This period of transition was also commented on by Singh-Carlson et al. [38] who identified many uncertainties regarding returning to work as well as concerns about the future to be included and examined as key elements of the SCP. What was common to all these authors was the potential for the SCP to identify particular psychosocial concerns as well as provide resources and supports that could be used by survivors and health professionals in the longer term.

Interestingly, Baravelli et al. [30] reported that the use of the SCP may also cause some distress to some survivors particularly when information regarding the recurrence of cancer was highlighted. This is a key point of interest for all people involved in the development of the SCP and one which warrants further exploration.

Several ideas regarding the most suitable format for the SCP were raised. Singh Carlson et al. [37] stressed the need for the SCP to be written in a language suitable for the population group and presented as a written, portable document so that survivors could use it as a key resource when negotiating new services or engaging other health professionals. Other studies indicated the need for a ‘living’ document available in electronic format [33, 35] which could be modified and readily available to all stakeholders.

There were varied views regarding who was most suited to develop the SCP. The primary care physician (PCP) or general practitioner (GP) was identified as suitable [29, 32, 34, 39], as were the oncology or specialist provider [33] and oncology nurse [36]. Other studies did not reach a clear consensus about who should take primary responsibility for this [30, 31, 38]. Only three studies stressed the need for survivors themselves to be included in the development of their own SCP [29, 36, 37].

Many studies identified a range of barriers associated with incorporating the SCP into their current model of practice including a lack of training available to assist health professionals to prepare these [31, 32, 34, 35] and the time required to develop and prepare SCPs [29, 32–34, 38]. Also noted by Faul et al. [33] and Hewitt et al. [34] was the uncertainty regarding responsibility for the cost of developing SCPs with many models of care not providing financial assistance for these additional resources.

### Cancer survivors do not consistently receive the SCP

There was a wide range of findings regarding availability and access to SCPs. Ashing-Giwa et al. [29] reported that only one of the 25 participants in their study had received a SCP. Other authors [34, 36] reported that ‘few to some’ had accessed the resource. Only one of all the reviewed studies [33] indicated a consistent provision of the SCP as part of the cancer survivorship period. Baravelli et al. [30] indicated that only one quarter of cancer survivors in their study received a written statement of any type regarding diagnosis. Ten percent had received a treatment summary and 15 % had received copies of diagnostic tests. While this information was recognised as important components of SCPs, it was also acknowledged that there was very little information provided regarding what to expect in the future such as long-term effects of treatment, potential psychosocial concerns and resources for ongoing problems. Four studies [31, 32, 37, 38] reported that this issue may be related to the varying models of practice, inconsistencies around the coordination of survivorship care and a lack of consensus regarding the most appropriate time to provide the SCP to survivors.

### Lack of evidence to support the use of SCP in practice

Adding to the potential reasons for why SCPs are not used routinely is the lack of clear evidence to support the use of the SCP in clinical practice. Recommendations were made by all authors regarding the need to conduct both qualitative and quantitative studies regarding the efficacy and application of SCPs in the future. Of considerable interest was the essential research needed to determine the opinions and preferences of cancer survivors themselves contributing to the research dialogue concerning SCPs [30, 34]. Two studies [33, 37] indicated a dearth of research regarding whether SCPs resulted in improved care and outcomes for cancer survivors. Additional statements by two authors [29, 32] stated that prospective longitudinal studies were vital for determining the long-term benefits and any added value of using SCPs as part of the overall care of cancer survivors.

Further recommendations were made to suggest that both qualitative and quantitative studies were needed to support if and how the SCP could be integrated into standard oncology care [33] and the health professionals best suited to provide them [31, 35, 38]. Parry et al. [37] argued that research was needed to evaluate each component of the SCP to substantiate their use across the various contexts of use.

## Discussion

This systematic review considered the range of data collected by the authors of 11 studies, all contributing to the qualitative evidence regarding the research question. According to the Institute of Medicine [8], the purpose of a SCP is to include a summary of cancer diagnosis and treatment, information regarding likely consequences of treatment and follow-up health information. It should also document information regarding health insurance, employment issues and psychosocial support. While the use of SCPs has varied amongst countries including the USA, Canada, UK and Australia, the availability and consistency of use is also not constant across the various contexts of care. According to the studies reviewed, there are a number of barriers and enablers influencing their acceptability and integration into the various models of care.

A key concern is that the survivorship period requires improved recognition as an integral period of the cancer journey. The reviewed studies support other literature regarding an emerging awareness and recognition of survivorship as a distinct part of the cancer journey [13, 41, 42]. It is apparent that health policies and the models of care that support cancer survivors also require attention so as to consider the many variables impacting this group of consumers [16, 24]. A consistent approach to the delivery of supportive services to cancer survivors, including the use of SCPs, is essential and must be prioritised for the future [6, 26, 32, 37].

According to the IOM [8], SCPs have the potential to empower and inform survivors about diagnosis and treatment, monitoring required and follow-up care available as well as act as a communication tool between stakeholders in order to maximise health [26]. However, greater consultation is needed between patients and the health professionals involved to ensure that the SCP is individualised and reflects the key concerns and issues for the cancer survivor. Cancer survivors are frequently not included in the development of the SCP, and therefore not targeted to the specific needs of individuals [3]. This is of particular concern as the need for consumers to be involved and ‘in charge’ of their health care requirements is regarded as an essential component of contemporary health practice [13, 16].

Further considerations for the development of SCPs might require adoption of a generic template with options for people with specific types of cancer and particular population groups. As noted by several authors, a much greater consideration of the psychosocial concerns experienced by cancer survivors is needed and included for discussion [9, 41, 43]. Some specific concerns include sexuality, intimacy, mood and adjustment to previous roles and relationships [6, 44, 45]. Additional educational information for financial, social, health and spiritual supports may also be required [8, 18, 46].

Agreement on many of the practical issues regarding use of the SCP is yet to be reached. Who is responsible for developing the SCP, what it should include and how it should be developed are still unclear. Some potential reasons for this may include time constraints, the cost of preparing the SCP and a lack of rigid evaluation regarding the efficacy of these [16, 18, 47, 48]. Also noted is the requirement to meet the needs of specific population groups, e.g. African-American, Southeast Asian and others with unique needs to address issues relevant to these cancer survivors.

Finally, there has been a growing effort by researchers to explore the effectiveness of SCPs for cancer survivors. In a recent systematic review conducted by Martin et al. [3], it concluded that while limited evidence existed regarding the effectiveness of SCP for a group of breast cancer survivors, the SCP did assist with the assessment and symptom management of survivors in the longer term. In contrast, other researchers concluded that the use of SCPs could assist health professionals to determine strategies for surveillance, increase communication amongst stakeholders and transition care from a medical model to a wellness model [17, 18, 20].

## Conclusion

This systematic review examined the experiences of cancer survivors using survivorship care plans and explored many of the current issues relating to their use across a range of different contexts. While the period of cancer survivorship is gaining interest amongst clinicians and researchers, it is clear that further studies are needed to explore the range of SCPs available, the practicalities related to their use and how to best ensure they meet the needs of cancer survivors in the future.

## Limitations

It is recognised that there are many published studies using quantitative methodologies in the subject area of SCPs and that these may offer additional data and discussion regarding the topic. This review also targeted the use of SCPs from the perspective of survivors and therefore the experiences of other key stakeholders (treating medical professionals and providers of support services) are not articulated.
